# Culture-Independent Microbiological Analysis of Foley Urinary Catheter Biofilms

**DOI:** 10.1371/journal.pone.0007811

**Published:** 2009-11-12

**Authors:** Daniel N. Frank, Shandra S. Wilson, Allison L. St. Amand, Norman R. Pace

**Affiliations:** 1 Department of Molecular, Cellular, and Developmental Biology, University of Colorado, Boulder, Colorado, United States of America; 2 Mucosal and Vaccine Research Program Colorado, University of Colorado, Denver, Colorado, United States of America; 3 Department of Urologic Oncology, University of Colorado Denver and Health Sciences Center, Aurora, Colorado, United States of America; Columbia University, United States of America

## Abstract

**Background:**

Prevention of catheter-associated urinary tract infection (CAUTI), a leading cause of nosocomial disease, is complicated by the propensity of bacteria to form biofilms on indwelling medical devices [1], [2], [3], [4], [5].

**Methodology/Principal Findings:**

To better understand the microbial diversity of these communities, we report the results of a culture-independent bacterial survey of Foley urinary catheters obtained from patients following total prostatectomy. Two patient subsets were analyzed, based on treatment or no treatment with systemic fluoroquinolone antibiotics during convalescence. Results indicate the presence of diverse polymicrobial assemblages that were most commonly observed in patients who did not receive systemic antibiotics. The communities typically contained both Gram-positive and Gram-negative microorganisms that included multiple potential pathogens.

**Conclusion/Significance:**

Prevention and treatment of CAUTI must take into consideration the possible polymicrobial nature of any particular infection.

## Introduction

Nosocomial urinary tract infections associated with catheterization occur in more than 1 million U.S. patients each year [4], [6], [7]. Although catheter-associated urinary tract infections (CAUTI) may not result in excess mortality [8], they significantly burden the health care system by increasing both morbidity and treatment costs [6]. Urinary catheter placement not only increases the access of potential pathogens to the bladder, but also provides a ready surface on which biofilms can form [1]. Microscopic observations show that catheter biofilm-associated bacteria form polymicrobial microcolonies that are embedded within an amorphous, protective extracellular matrix [5], [9], [10], [11], [12]. Treatment is complicated by the resistance of biofilm-associated microorganisms to antibiotics that are otherwise effective in treating cells in the planktonic state [9].

Diverse microbial species have been identified from catheter biofilms by microbiological culture [5], [13], [14], [15]. However, Nickel et al [10] reported that although multiple morphological types were observed to colonize Foley urinary catheters, only a small fraction of the attendant microorganisms could be detected by traditional microbiological culture. Hence, the identities of microorganisms that participate in catheter biofilm formation and maintenance may not be fully determined. To complement previous results based on traditional microbial culture (e.g. Barford et. al., [15]), we have used rRNA-based molecular-phylogenetic methods that do not require prior culture [16], [17] to comprehensively survey the types of microorganisms that colonize Foley urinary catheters.

## Results

Foley catheters were obtained from 14 patients catheterized for two weeks following total prostatectomy. Two patient subsets were analyzed, based on treatment (n = 6) or no treatment (n = 8) with systemic fluoroquinolone antibiotics during convalescence. Patients were randomly assigned to treatment groups independently of this study.

**Figure 1:**
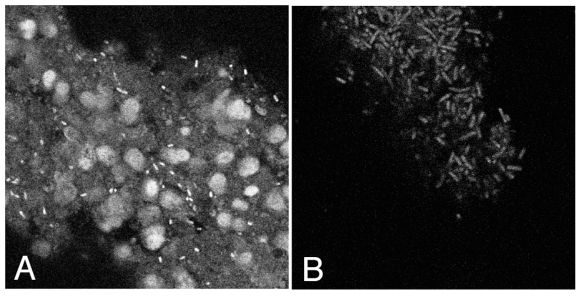
Confocal micrographs of catheter biofilm. Material scraped from the outside (panel A) and inside (panel B) surface of catheter sample Cat01 was stained with DAPI and visualized by confocal microscopy. Micrographs were taken at 100X magnification; nuclei in panel A, probably of human origin, indicate the relative sizes of bacteria.

The section of a Foley catheter that is inserted into a bladder consists of a hollow, cylindrical rubber tube that terminates with a solid rubber tip. A hole formed latterly through the catheter allows fluids to drain into the internal cavity of the catheter tubing and thereby flow out of the bladder. Upon removal from a bladder, each catheter was sliced in half longitudinally using a sterile scalpel. Biomass was removed from the internal cavities and external catheter surfaces by forcefully scraping material from each location into a microcentrifuge tube with a sterile scalpel (Materials and Methods). Additionally, the tip of each catheter (∼5 mm in length) was excised with a scalpel and placed in a sterile microcentrifuge tube. Confocal microscopic analysis of DAPI-stained catheter biomass revealed the presence of dense matrices of microbial cells (examples shown in Fig. 1), similar to those observed through electron microscopy [10], [11], [18]. Eucaryotic cells, probably of human origin due to their epithelium-like cell morphology, also were observed in several samples prepared from outer catheter surfaces. We did not survey eukaryotes by broad-range PCR and, therefore, cannot rule out the presence of fungi or other eucaryal cells.

Mixed community genomic DNA was prepared from each specimen by boiling in buffer containing non-ionic detergent (TE + NP40) followed by freezing at −20C. Previous studies indicate that this protocol disrupts a wide range of cell-types, including Gram-positive and Gram-negative bacteria, actinobacteria, and fungi [19], [20].

Microorganisms present in the specimens were identified by molecular-phylogenetic analysis of 16S ribosomal RNA gene (rDNA) sequences, following broad-range PCR amplification of rDNA from the DNA lysates. The primer set that was used, 8F/907R, is broadly specific for all known bacteria. Broad-range rDNA amplification was successful in 16.6% (3/18) of antibiotic treated and 79% (19/24) of untreated specimens (Table 1). PCR results were discordant between the inside, outside, and tip samples for only two catheters (Cat01 and Cat03); in both cases no PCR product was obtained from material collected from the catheter interiors. Individual rDNA clone libraries were constructed for all 22 PCR positive catheter specimens and a total of 1763 rDNA sequences determined.

**Table 1:** Results of broad-range rDNA PCR.

Specimen	Antibiotic	Site	PCR
Cat01	No	Exterior	**+**
		Tip	**+**
		Interior	**−**
Cat02	No	Exterior	**−**
		Tip	**−**
		Interior	**−**
Cat03	No	Exterior	**+**
		Tip	**+**
		Interior	**−**
Cat04	No	Exterior	**+**
		Tip	**+**
		Interior	**+**
Cat05	No	Exterior	**+**
		Tip	**+**
		Interior	**+**
Cat06	No	Exterior	**+**
		Tip	**+**
		Interior	**+**
Cat07	No	Exterior	**+**
		Tip	**+**
		Interior	**+**
Cat08	No	Exterior	**+**
		Tip	**+**
		Interior	**+**
Cat09	Yes	Exterior	**−**
		Tip	**−**
		Interior	**−**
Cat10	Yes	Exterior	**−**
		Tip	**−**
		Interior	**−**
Cat11	Yes	Exterior	**−**
		Tip	**−**
		Interior	**−**
Cat12	Yes	Exterior	**−**
		Tip	**−**
		Interior	**−**
Cat13	Yes	Exterior	**+**
		Tip	**+**
		Interior	**+**
Cat14	Yes	Exterior	**−**
		Tip	**−**
		Interior	**−**

Following their alignment [21], rDNA sequences were clustered into operational taxonomic units (OTUs) of approximately species-level taxonomic rank, defined as relatedness groups with mean pairwise distances of <3%. Sampling statistics (Table 2) indicated that, taken together, the multiple clone libraries constructed for each catheter were representative of the microbial populations that colonized it: Good's coverage values were in the range of 83%–100% (mean 97%). The least OTU-rich specimen was obtained from a patient treated with systemic antibiotics, suggesting that this medical treatment suppressed the diversity of potential colonizing microbes. In contrast, antibiotic non-treatment was associated with polymicrobial colonization of catheters. The non-parametric OTU-richness estimator S_chao1_ indicated that these catheters were colonized by an average of 22.7 species-level groups (range of 3 to 68; Table 2).

**Table 2:** Sampling coverage and richness estimates for 97% OTUs.

Specimen	N^1^	Sobs^2^	Good^3^	Schao1^4^	LCI95^5^	UCI95^5^	Antibiotic
Cat01	156	3	99%	3	3	3	−
Cat03	104	33	83%	55	40	101	−
Cat04	236	47	92%	68	54	112	−
Cat05	278	7	100%	7	7	7	−
Cat06	276	10	99%	11	10	18	−
Cat07	265	8	99%	9	8	22	−
Cat08	276	6	100%	6	6	6	−
Cat13	172	1	100%	1	1	1	+

1 Sequences analyzed.

2 Observed OTUs in all samples from catheter.

3 Good's coverage estimate.

4 Bias-corrected Chao1 estimate of OTU richness.

5 LCI: lower 95% confidence interval. UCI: upper 95% confidence interval.

Microorganisms present in catheter biofilms were identified by BLASTN [22] search of rDNA sequence databases and molecular-phylogenetic analysis. The predominant types of microorganisms identified by rRNA analysis and their relative frequencies are summarized in Table 3 (data are presented in full in Table S1). BLAST percent identities and bit scores were quite high between catheter sequences and their top BLAST hits. Few sequences were 100% identical to one another or to previously reported sequences, indicating a high level of sequence microheterogeneity among the catheter colonizing microbial communities. Nevertheless, 99.5% (1755/1763) of sequences were at least 97% identical to their top BLAST hits, meaning that nearly all sequences could be assigned to genus and species with high confidence. All sequences were >93% identical to at least one rDNA sequence in GenBank.

**Table 3:** Distribution of bacterial rDNA sequences in catheter samples.

	Catheter Specimens:^1^	Cat01	Cat03	Cat04	Cat05	Cat06	Cat07	Cat08	Cat13		
	Antibiotic Treatment:^2^	−	−	−	−	−	−	−	+		
**Top BLAST Hit** 3	**%ID** 4									**N** 5	**Prevalence**
**Proteobacteria**											
*Pseudomonas aeruginosa*	99	0	7.7	0	1.1	0	**32.8**	15.9	**100.0**	314	63%
*Klebsiella pneumoniae*	99	**94.9**	0	1.3	2.2	**29.0**	0	24.3	0	304	63%
*Escherichia coli*	99	0	**13.5**	3.4	1.1	18.5	0	0.0	0	76	50%
* Delftia tsuruhatensis*	99	0	0	2.1	0	11.6	0	0	0	37	25%
* Acinetobacter lwoffii*	99	0	0	0	0	11.6	0	0	0	32	13%
*Enterobacter hormaechei*	99	0	0	0	0	0	0	10.5	0	29	13%
Sphingomonas sp.	99	0	0	0	0	10.1	0	0	0	28	13%
Klebsiella sp. HF2	98	0	0	0	0	8.0	0	0	0	22	13%
Oxalobacteraceae sp.	98	0	1.0	6.4	0	0	0	0	0	16	25%
* Achromobacter xylosoxidans*	99	0	6.7	3.0	0	0	0	0	0	14	25%
* Burkholderia fungorum*	98	0	0.0	5.9	0	0	0	0	0	14	13%
**Firmicutes**											
* Staphylococcus epidermidis*	99	0	0	**31.4**	0	0	30.6	0	0	155	25%
* Streptococcus pneumoniae*	99	0	**13.5**	3.0	0	0	0	**33.0**	0	112	38%
*Enterococcus faecalis*	99	0	0	0	**37.8**	0	0	0.0	0	105	13%
*Enterococcus faecalis*	98	0	0	0	29.1	0	0	0.0	0	81	13%
* Anaerococcus vaginalis*	98	0	0	0	28.1	0	0	0.0	0	78	13%
* Peptoniphilus asaccharolyticus*	99	0	1.0	0	0	0	0	15.9	0	45	25%
* Finegoldia magna*	99	0	1.0	0	0	0	16.2	0	0	44	25%
**Bacteroidetes**											
* Porphyromonas somerae*	89	0	0	0	0	0	16.2	0	0	43	13%
Flavobacterium sp.	98	0	0	4.2	0	0	0	0	0	10	13%
**Nitrospira**											
* Nitrospira marina*	88	0	0	0	0	9.8	0	0	0	27	13%
**Actinobacteria**											
* Mycobacterium intracellulare*	99	0	**13.5**	0	0	0	0	0	0	14	13%
	Other:^6^	5.1	42.3	39.4	0.7	1.4	4.2	0.4	0.0	9.2	
	Total Clones:^5^	156	104	236	278	276	265	276	172	1763	

1 Numbers designate the percentage of clones in a catheter library that were classified in a phylotype. High values for each specimen are in bold.

2 Designates whether a patient was subjected to systemic antibiotics during catheterization.

3 Identified by Blast search of database culled of environmental clones.

4 Mean BLAST percent identity scores of query sequences to top BLAST hit.

5 Total number of sequences.

6 Low abundance clones were removed from table because of space limitations. See Table S1.

**Figure 2:**
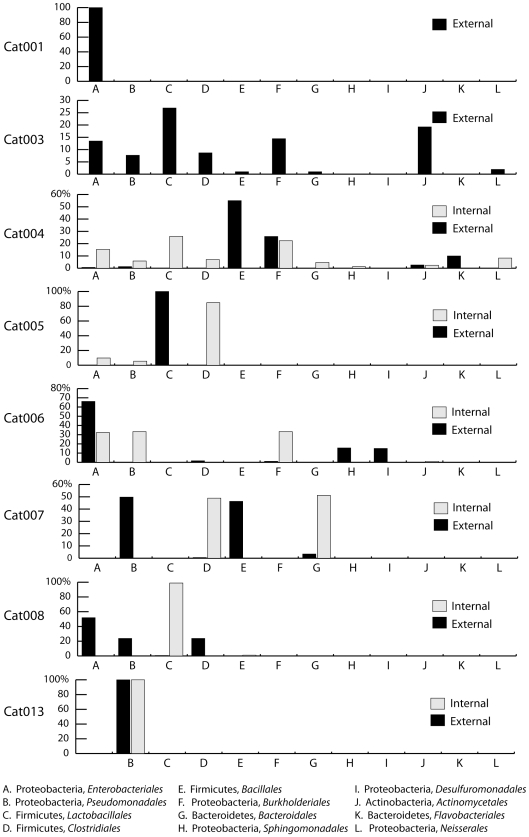
Phylogenetic distribution of 16S rRNA clones recovered from internal and external catheter surfaces. Each barchart compares the taxonomic order-level distributions of sequences isolated from internal and external catheter specimens. Lower abundance bacterial orders (i.e., those with clonal representation less than 10% of total sequences) are not shown.

Overall, members of the bacterial phyla *Proteobacteria* (54% of clones) and *Firmicutes* (38% of clones) dominated the rDNA clone libraries. Five of seven antibiotic-naïve catheters harbored members of both phyla. At the species level, the most abundant phylotypes were representative of microorganisms that colonize and/or infect humans, including *Pseudomonas aeruginosa, Klebsiella pneumoniae, Staphylococcus epidermidis, Streptococcus pneumoniae*, *Escherichia coli, Enterococcus faecalis,* and *Propionibacterium acnes.* A recent, culture-dependent analysis of catheter biofilms also reported the identification of similar microorganisms, including *Staphylococcus epidermidis, Enterococcus faecalis, Pseudomonas spp,* and *Klebsiella spp* [15]. Many taxa were identified in multiple catheters (prevalences ranged from 13%–63%). Moreover, at least two potential pathogens were identified in the majority of (6/7) of catheters not exposed to systemic antibiotics (e.g., *P. aeruginosa. K. pneumoniae, E. coli,* and *E. faecalis* all were detected on specimen Cat05). Extensive variation also was evident between the different sites sampled on each catheter. This can be seen in Figure 2, which shows the distributions of rRNA sequences, organized at the level of taxonomic order, recovered from the external and internal catheter surfaces (Table S1 provides species-level data).

## Discussion

This study provides the first culture-independent examination of the types of microorganisms that colonize Foley urinary catheters. Most organisms that were encountered were closely related to known human opportunistic or true pathogens. These microbes usually were observed in association with multiple other species, including other potentially pathogenic organisms. All have been cultured from catheters [5], [13], [14] and commonly are associated with nosocomial infection, in agreement with the hypothesis that biofilm formation on Foley catheter surfaces is a first step leading to urinary tract infections.

Comparison of individual specimens revealed that catheter biofilm communities are not characterized by a common species composition. Instead, a unique suite of microbial groups composed of multiple genera and phyla of bacteria colonized each catheter. Although in several cases the catheter tips and exteriors were similar in community structure (e.g. Cat01, Cat03, Cat05, Cat06, Cat13), catheter interiors often were substantially different from their exteriors at the species level (Table S1). Catheters were washed in phosphate-buffered saline prior to biofilm collection so it is unlikely that these differences resulted from urethral contamination upon extraction of the catheters. Rather, as suggested by previous ultrastructural studies [10], the distribution of microorganisms on a catheter surface is likely to consist of patchy, distinct microcolonies.

In summary, a variety of potential pathogens, many previously associated with CAUTI, were identified by culture-independent analysis of Foley catheters. Microorganisms were most often observed in polymicrobial communities that included multiple potential pathogenic species from different phyla. Thus, treatment and prevention of CAUTI must take into consideration the diverse populations of UTI-causing microorganisms that may co-inhabit any catheter.

## Materials and Methods

### Patients

Foley urinary catheters were collected from patients following radical prostatectomy surgery, performed at University of Colorado Denver and Health Sciences Center. Independent of this research project, patients were randomly chosen to receive antibiotics (500 mg ciprofloxacin orally twice a day) or not. Catheters were removed two-weeks after surgery and were collected sequentially from treated and untreated patients. All samples were deidentified. The protocol was approved by the Institutional Review Board of the University of Colorado, Boulder for analysis of deidentified medical waste.

### Sample Acquisition, Storage, and Transport

Upon removal from the patients' urethras, catheters were stored at −20°C in a sterile solution of 10% H2O, 70% Ethanol, and 20% buffer-saturated Phenol (pH 8; Sigma-Aldrich, St. Louis, USA), and transported on wet ice to Boulder, CO for processing.

### Biomass Collection and DNA Extraction

All steps were performed in a laminar flow hood that was decontaminated by ultraviolet light. Samples of biomass were collected from three sites on each catheter: the internal cavity, external body, and tip. Catheters were rinsed briefly in phosphate buffered saline (PBS, Sigma-Aldrich, St. Louis, USA) in order to remove storage buffer and any loosely adhering microbes (for instance bacteria that became associated with catheters during removal through the urethra) and then placed in sterile Petri dishes. The tip of each catheter (∼5 mm in length) was excised with a sterile scalpel and placed in 200 µl of TE/NP40 in a sterile microcentrifuge tube. Catheters were then bisected longitudinally and biomass was removed from internal cavities and external bodies by forcefully scraping each site with a scalpel, rinsing the blade with 200 µl of TE/NP40 (10 mM Tris-pH 8.0/1 mM EDTA/0.1% NP40), and then collecting the buffer and biomass in separate, sterile microcentrifuge tubes. Additional material was collected for microscopy using the same technique, but TE was substituted for TE/NP40.

Cellular material was lysed and crude mixed-community genomic DNA prepared from each specimen by two 10-minute boiling steps followed by freezing at −20°C. Samples were then thawed and microcentrifuged for 15 minutes (13,000 rpm) to clear DNA lysates of cellular debris; cleared lysates were submitted directly to PCR without further purification. All DNA extraction and PCR amplification was conducted by D.N.F. in the laboratory of N.R.P. Although samples were not processed simultaneously, aliquoted and frozen reagents were used for DNA extractions and PCR amplifications in order to minimize sample-to-sample variation. Reagent-only, negative-extraction controls were included in each batch of DNA-extractions.

### rRNA Gene Library Construction

SSU rRNA genes were amplified from DNA samples by PCR with primers specific for all bacterial SSU rRNA genes: 8F (5′AGAGTTTGATCCTGGCTCAG) and 907R (5′CCGTCAATTCCTTTRAGTTT). Each 30 ml PCR reaction included 3 ml 10x PCR Buffer, 2.25 ml dNTP mix (2.5 mM each dNTP), 1.5 ml 50 mM MgCl_2_, 37.5 ng of each primer, 1.5 ml genomic DNA lysate, and 1 unit Taq polymerase, (Biolase polymerase; Bioline USA Inc., Boston, USA). Negative-extraction and no-template controls were run in parallel with each set of PCR reactions in order to control for exogenous contamination. Initially, all samples were subjected to 27 amplification cycles. If insufficient product was generated under these conditions to permit cloning of a particular sample, the PCR reaction was repeated with a protocol incremented by two cycles. Samples that failed to amplify after incrementing to 34 cycles were judged to be negative. No correlations were evident between the number of cycles needed to amplify product and the composition of the rRNA sequence libraries that were constructed.

PCR products were visualized under low-wavelength UV irradiation following agarose gel electrophoresis (1.5% agarose gel in tris-borate EDTA stained with ethidium bromide). PCR reactions were judged to be positive only if bands of the appropriate size were visible. Agarose gel slices encompassing all positive bands of a particular sample were excised with a sterile razor blade (a new blade was used for each sample). DNA was purified using the QIAquick® gel extraction kit (Qiagen Inc., Valencia, CA). Genes were cloned into the pCR4®-TOPO® vector of the Invitrogen TOPO® TA Cloning kit and transformed into One Shot TOP 10 competent cells following the manufacturer's instructions (Invitrogen Corp., Carlsbad, CA).

For each clone library, 96 transformants were grown overnight at 37°C in a 96-well culture plate filled with 1.5 mls of 2xYT medium per well. 20 ml of each overnight culture was added to 20 ml of 10 mM Tris-HCl (pH 8.0), heated 10 minutes at 95C, and centrifuged 10 minutes at 4000 rpm in a 96-well plate centrifuge (Eppendorf Inc., Westbury, NY). One ml of culture supernatant was used as template in a 30 ml PCR reaction with vector specific primers (T7 and T3 sites). Ten ml of each PCR product were first treated with the ExoSap-IT kit (USB Corp, Cleveland, OH) and then subjected to cycle sequencing with the Big-Dye Terminator kit (Applied Biosystems, Inc., Foster City, CA) following the manufacturers' protocols. Sequencing was performed on MegaBACE 1000 (Amersham Biosciences, Piscataway, NJ) automated DNA sequencers.

### Sequence Analysis

Sequence base calling and contig assembly were performed with the applications phred and phrap [23], [24], as implemented by XplorSeq [25]. Vector and primer sequences were removed along with flanking nucleotides of poor quality (Q<20). Initial microbial species identifications were made by a batch BLAST search of both GenBank and a local database of rRNA sequences culled of environmental/uncultured sequences using the client applications blastcl3 and blastall (NCBI). Sequences with BLAST bit scores lower than 400 and/or lengths less than 400 nucleotides were discarded. Potentially chimeric sequences, as suggested by either Bellerophon [26], also were removed from the dataset. Cloned sequences were aligned to an existing database of rRNA gene sequences [27] using the NAST alignment algorithm [21]. Taxonomic lineages were assigned following phylogenetic analyses using the application ARB [28]. Sequences were deposited in GenBank and assigned the accession numbers EU571960 - EU572707.

### Operational Taxonomic Units

Sequences were clustered into relatedness-groups (OTUs) by average-linkage clustering, using the application sortx [25]. Phylogenetic distances were uncorrected and calculated using only conserved positions specified by the Lane-mask [29]. A dataset was created by clustering sequences with a distance threshold of 97%. Sampling completeness was assessed by Good's Coverage estimator [30] and estimates of species richness were calculated using the non-parametric estimators ACE (abundance-based coverage estimator [31]) and Chao1 [32].

### Microscopy

Ten µl of biomass in TE were added to 0.5 ml 4 M paraformaldehyde and fixed overnight at 4°C. Solid material was then pelleted by microcentrifugation, washed twice in PBS, pelleted again by microcentrifugation, and finally resuspended in 0.1 ml of 70% ethanol. Ten µl of fixed cells were placed on a silane-coated microscope slide (Sigma-Aldrich) within a frame-seal (MJ Research, Inc.) and allowed to air dry. Sixty-five µl of a 1 µg/ml solution of 4′,6′-diamidino-2-phenylindole (DAPI) were then added to the fixed cells and allowed to incubate in the dark for 5 minutes. Microscope slides were then rinsed in Coplin jars filled with PBS, dipped briefly in cold 20 mM Tris pH 8 in order to remove excess salt and then mounted with Citifluor antifading reagent. Slides were visualized under epifluorescence using either a Nikon Eclipse E600 microscope or a Leica laser scanning confocal microscope

## Supporting Information

Table S1 Phylogenetic distribution of Foley catheter bacteria across sample sites.
(0.10 MB PDF)
