# Supplementary material for: Culture-Independent Microbiological Analysis of Foley Urinary Catheter Biofilms
Source: PLoS One. 2009 Nov 12;4(11):e7811. doi: 10.1371/journal.pone.0007811 (PMC2771765; doi:10.1371/journal.pone.0007811)
Supplement: Table S1 — Phylogenetic distribution of Foley catheter bacteria across sample sites. (0.10 MB PDF) [file pone.0007811.s001.pdf]

Table S1. Phylogenetic Distribution of Foley Catheter Bacteria across Sample Sites.

			Catheter Specimen																								
			Cat01I	Cat01J	Cat03E	Cat03F	Cat04B	Cat04C	Cat04D	Cat05F	Cat05G	Cat05H	Cat06F	Cat06G	Cat06H	Cat07B	Cat07C	Cat07D	Cat08B	Cat08C	Cat08D	Cat13B	Cat13C	Cat13D	Total Clones	Prev-alence	Entrez lineage of Top BLAST Hit
		Antibiotic Treatment:	-	-	-	-	-	-	-	-	-	-	-	-	-	-	-	-	-	-	-	+	+	+			
		Sample Site:	Ext	Tip	Ext	Tip	Ext	Tip	Int	Ext	Tip	Int	Ext	Tip	Int	Ext	Tip	Int	Ext	Tip	Int	Ext	Tip	Int			
**Top BLAST Hit**	**Accession**	**%ID**																									
**Proteobacteria**																											
Pseudomonas aeruginosa	EU099379	99	0	0	2.56	23.08	0	0	0	0	0	3.26	0	0	0	1.16	96.63	0	47.31	0	0	100	100	100	314	40.9	Proteobacteria; Gammaproteobacteria; Pseudomonadales;
Klebsiella pneumoniae	AY292865	99	100	90	0	0	0	0	3.53	0	0	6.52	5.49	49.44	32.29	0	0	0	5.38	67.39	0	0	0	0	304	40.9	Proteobacteria; Gammaproteobacteria; Enterobacteriales;
Escherichia coli	EU009184	99	0	0	14.1	11.54	0	0	9.41	0	0	3.26	31.87	24.72	0	0	0	0	0	0	0	0	0	0	76	27.3	Proteobacteria; Gammaproteobacteria; Enterobacteriales;
Delftia tsuruhatensis	EF421404	99	0	0	0	0	0	0	5.88	0	0	0	0	0	33.33	0	0	0	0	0	0	0	0	0	37	9.1	Proteobacteria; Betaproteobacteria; Burkholderiales;
Acinetobacter lwoffii	DQ279314	99	0	0	0	0	0	0	0	0	0	0	0	0	33.33	0	0	0	0	0	0	0	0	0	32	4.5	Proteobacteria; Gammaproteobacteria; Pseudomonadales;
Enterobacter sp. '05 01 120'	EF088367	99	0	0	0	0	0	0	0	0	0	0	0	0	0	0	0	0	0	31.52	0	0	0	0	29	4.5	Proteobacteria; Gammaproteobacteria; Enterobacteriales;
Sphingomonas sp. DhA-95	AF177917	99	0	0	0	0	0	0	0	0	0	0	30.77	0	0	0	0	0	0	0	0	0	0	0	28	4.5	Proteobacteria; Alphaproteobacteria; Sphingomonadales;
Klebsiella sp. HF2	DQ998840	98	0	0	0	0	0	0	0	0	0	0	0	24.72	0	0	0	0	0	0	0	0	0	0	22	4.5	Proteobacteria; Gammaproteobacteria; Enterobacteriales;
Oxalobacteraceae bacterium QD1	DQ388765	98	0	0	0	3.85	0	11.96	4.71	0	0	0	0	0	0	0	0	0	0	0	0	0	0	0	16	13.6	Proteobacteria; Betaproteobacteria; Burkholderiales;
Achromobacter xylosoxidans	DQ414679	99	0	0	7.69	3.85	0	2.17	5.88	0	0	0	0	0	0	0	0	0	0	0	0	0	0	0	14	18.2	Proteobacteria; Betaproteobacteria; Burkholderiales;
Burkholderia fungorum	EF650018	98	0	0	0	0	0	15.22	0	0	0	0	0	0	0	0	0	0	0	0	0	0	0	0	14	4.5	Proteobacteria; Betaproteobacteria; Burkholderiales;
Klebsiella sp. NB5	AM269473	99	0	8.75	0	0	0	0	2.35	0	0	0	0	0	0	0	0	0	0	0	0	0	0	0	9	9.1	Proteobacteria; Gammaproteobacteria; Enterobacteriales;
Neisseria sp. R-22841	AJ786809	99	0	0	2.56	0	0	0	8.24	0	0	0	0	0	0	0	0	0	0	0	0	0	0	0	9	9.1	Proteobacteria; Betaproteobacteria.
Aquaspirillum autotrophicum	AB074524	99	0	0	1.28	0	0	6.52	0	0	0	0	0	0	0	0	0	0	0	0	0	0	0	0	7	9.1	Proteobacteria; Betaproteobacteria; Burkholderiales;
Pseudomonas mephitica	AM748811	99	0	0	7.69	0	0	0	1.18	0	0	0	0	0	0	0	0	0	0	0	0	0	0	0	7	9.1	Proteobacteria; Betaproteobacteria; Burkholderiales;
Acinetobacter sp. RBE2CD-28	EF111219	99	0	0	0	0	0	2.17	2.35	0	0	0	0	0	0	0	0	0	0	0	0	0	0	0	4	9.1	Proteobacteria; Gammaproteobacteria; Pseudomonadales;
Marinomonas sp. BSi20487	EF382679	98	0	0	0	3.85	0	3.26	0	0	0	0	0	0	0	0	0	0	0	0	0	0	0	0	4	9.1	Proteobacteria; Gammaproteobacteria; Oceanospirillales;
Variovorax sp. 44/31	AY571831	99	0	0	0	0	0	4.35	0	0	0	0	0	0	0	0	0	0	0	0	0	0	0	0	4	4.5	Proteobacteria; Betaproteobacteria; Burkholderiales;
Pseudomonas sp. ONBA-17	DQ079062	99	0	0	0	0	0	0	3.53	0	0	0	0	0	0	0	0	0	0	0	0	0	0	0	3	4.5	Proteobacteria; Gammaproteobacteria; Pseudomonadales;
Massilia timonae strain 99A9205	AY157761	99	0	0	0	0	0	0	3.53	0	0	0	0	0	0	0	0	0	0	0	0	0	0	0	3	4.5	Proteobacteria; Betaproteobacteria; Burkholderiales;
Stenotrophomonas sp. JRL-2	AY747593	99	0	0	0	3.85	0	0	1.18	0	0	0	0	0	0	0	0	0	0	0	0	0	0	0	2	9.1	Proteobacteria; Gammaproteobacteria; Xanthomonadales;
Stenotrophomonas sp. 2R13	EF178465	99	0	0	0	0	0	0	2.35	0	0	0	0	0	0	0	0	0	0	0	0	0	0	0	2	4.5	Proteobacteria; Gammaproteobacteria; Xanthomonadales;
Pseudomonas sp. OW5-19-6	EF523609	99	0	0	0	0	0	0	0	0	0	2.17	0	0	0	0	0	0	0	0	0	0	0	0	2	4.5	Proteobacteria; Gammaproteobacteria; Pseudomonadales;
Ralstonia sp. K101	AB212229	92	0	0	0	0	0	0	0	0	0	0	2.2	0	0	0	0	0	0	0	0	0	0	0	2	4.5	Proteobacteria; Betaproteobacteria; Burkholderiales;
Soil bacterium TWE165	DQ493433	99	0	0	0	0	0	2.17	0	0	0	0	0	0	0	0	0	0	0	0	0	0	0	0	2	4.5	Proteobacteria; Alphaproteobacteria.
Nevskia ramosa	AJ001010	99	0	0	1.28	0	0	0	0	0	0	0	0	0	0	0	0	0	0	0	0	0	0	0	1	4.5	Proteobacteria; Gammaproteobacteria; Xanthomonadales;
Providencia sp. OP1	AM040495	99	0	0	0	0	0	1.09	0	0	0	0	0	0	0	0	0	0	0	0	0	0	0	0	1	4.5	Proteobacteria; Gammaproteobacteria; Enterobacteriales;
Klebsiella pneumoniae	AY292866	97	0	1.25	0	0	0	0	0	0	0	0	0	0	0	0	0	0	0	0	0	0	0	0	1	4.5	Proteobacteria; Gammaproteobacteria; Enterobacteriales;
Variovorax sp. 44/31	AY571831	99	0	0	0	0	0	1.09	0	0	0	0	0	0	0	0	0	0	0	0	0	0	0	0	1	4.5	Proteobacteria; Betaproteobacteria; Burkholderiales;
Massilia aerolata	EF688526	99	0	0	0	0	0	0	1.18	0	0	0	0	0	0	0	0	0	0	0	0	0	0	0	1	4.5	Proteobacteria; Betaproteobacteria; Burkholderiales;
Aquaspirillum autotrophicum	AB074524	99	0	0	0	0	0	1.09	0	0	0	0	0	0	0	0	0	0	0	0	0	0	0	0	1	4.5	Proteobacteria; Betaproteobacteria; Burkholderiales;
Burkholderia symbiont of A. aquaticus	AY447042	99	0	0	0	0	0	1.09	0	0	0	0	0	0	0	0	0	0	0	0	0	0	0	0	1	4.5	Proteobacteria; Betaproteobacteria; Burkholderiales;
Sphingopyxis sp. 1_1K	EF540445	99	0	0	0	0	0	0	1.18	0	0	0	0	0	0	0	0	0	0	0	0	0	0	0	1	4.5	Proteobacteria; Alphaproteobacteria; Sphingomonadales;
Bradyrhizobium sp. Is-D302	EF435003	98	0	0	0	0	0	0	1.18	0	0	0	0	0	0	0	0	0	0	0	0	0	0	0	1	4.5	Proteobacteria; Alphaproteobacteria; Rhizobiales;
**Firmicutes**																											
Staphylococcus epidermidis	EU071604	99	0	0	0	0	98.31	17.39	0	0	0	0	0	0	0	94.19	0	0	0	0	0	0	0	0	155	13.6	Firmicutes; Bacillales; Staphylococcus.
Streptococcus pneumoniae	NZ ABAF01000005	99	0	0	14.1	11.54	0	0	8.24	0	0	0	0	0	0	0	0	0	0	1.09	98.9	0	0	0	112	22.7	Firmicutes; Lactobacillales; Streptococcaceae;
Enterococcus faecalis	AB292313	99	0	0	0	0	0	0	0	67.02	45.65	0	0	0	0	0	0	0	0	0	0	0	0	0	105	9.1	Firmicutes; Lactobacillales; Enterococcaceae;
Enterococcus faecalis	AB292313	98	0	0	0	0	0	0	0	32.98	54.35	0	0	0	0	0	0	0	0	0	0	0	0	0	81	9.1	Firmicutes; Lactobacillales; Enterococcaceae;
Anaerococcus vaginalis	AF542229	98	0	0	0	0	0	0	0	0	0	84.78	0	0	0	0	0	0	0	0	0	0	0	0	78	4.5	Firmicutes; Clostridia; Clostridiales;
Peptoniphilus asaccharolyticus	DQ986463	99	0	0	1.28	0	0	0	0	0	0	0	0	0	0	0	0	0	47.31	0	0	0	0	0	45	9.1	Firmicutes; Clostridia; Clostridiales;
Finegoldia magna	AB109769	99	0	0	0	3.85	0	0	0	0	0	0	0	0	0	0	0	47.78	0	0	0	0	0	0	44	9.1	Firmicutes; Clostridia; Clostridiales;
Streptococcus gordonii	AY485606	98	0	0	3.85	0	0	0	7.06	0	0	0	0	0	0	0	0	0	0	0	0	0	0	0	9	9.1	Firmicutes; Lactobacillales; Streptococcaceae;
Bacillus sp. 8-1	AB043842	99	0	0	0	0	0	9.78	0	0	0	0	0	0	0	0	0	0	0	0	0	0	0	0	9	4.5	Firmicutes; Bacillales; Bacillaceae; Bacillus.
Streptococcus parasanguis	AM157441	99	0	0	5.13	3.85	0	0	0	0	0	0	0	0	0	0	0	0	0	0	0	0	0	0	5	9.1	Firmicutes; Lactobacillales; Streptococcaceae;
Streptococcus salivarius	AY188352	99	0	0	1.28	0	0	0	4.71	0	0	0	0	0	0	0	0	0	0	0	0	0	0	0	5	9.1	Firmicutes; Lactobacillales; Streptococcaceae;
Streptococcus anginosus	DQ232518	99	0	0	1.28	3.85	0	0	2.35	0	0	0	0	0	0	0	0	0	0	0	0	0	0	0	4	13.6	Firmicutes; Lactobacillales; Streptococcaceae;
Streptococcus equi	EF406015	98	0	0	2.56	0	0	0	1.18	0	0	0	0	0	0	0	0	0	0	0	0	0	0	0	3	9.1	Firmicutes; Lactobacillales; Streptococcaceae;
Streptococcus sanguinis	AY691542	99	0	0	0	0	0	0	2.35	0	0	0	0	0	0	0	0	0	0	0	0	0	0	0	2	4.5	Firmicutes; Lactobacillales; Streptococcaceae;
Peptococcus niger	X55797	99	0	0	0	7.69	0	0	0	0	0	0	0	0	0	0	0	0	0	0	0	0	0	0	2	4.5	Firmicutes; Clostridia; Clostridiales; Peptococcaceae;
Veillonella dispar	AY995770	98	0	0	0	0	0	0	2.35	0	0	0	0	0	0	0	0	0	0	0	0	0	0	0	2	4.5	Firmicutes; Clostridia; Clostridiales;
Anaerococcus prevotii	AF542232	98	0	0	0	7.69	0	0	0	0	0	0	0	0	0	0	0	0	0	0	0	0	0	0	2	4.5	Firmicutes; Clostridia; Clostridiales;
Desemzia incerta	EF491964	99	0	0	1.28	0	0	0	0	0	0	0	0	0	0	0	0	0	0	0	0	0	0	0	1	4.5	Firmicutes; Lactobacillales; Carnobacteriaceae; Desemzia.
Catonella morbi	X87151	97	0	0	0	0	0	0	1.18	0	0	0	0	0	0	0	0	0	0	0	0	0	0	0	1	4.5	Firmicutes; Clostridia; Clostridiales; Lachnospiraceae;
Lachnospiraceae genomosp. C1	AY278618	99	0	0	0	0	0	0	1.18	0	0	0	0	0	0	0	0	0	0	0	0	0	0	0	1	4.5	Firmicutes; Clostridia; Clostridiales; Lachnospiraceae;
Anaerococcus prevotii	AF542232	98	0	0	0	0	0	0	0	0	0	0	0	0	0	1.16	0	0	0	0	0	0	0	0	1	4.5	Firmicutes; Clostridia; Clostridiales;
Peptoniphilus sp. WAL 10418	DQ911241	96	0	0	0	3.85	0	0	0	0	0	0	0	0	0	0	0	0	0	0	0	0	0	0	1	4.5	Firmicutes; Clostridia; Clostridiales;
Megasphaera genomosp. C1	AY278622	99	0	0	0	0	0	0	1.18	0	0	0	0	0	0	0	0	0	0	0	0	0	0	0	1	4.5	Firmicutes; Clostridia; Clostridiales;
Selenomonas flueggei	AF287803	96	0	0	0	0	0	0	1.18	0	0	0	0	0	0	0	0	0	0	0	0	0	0	0	1	4.5	Firmicutes; Clostridia; Clostridiales;
Anaerococcus vaginalis	AF542229	99	0	0	0	0	0	0	0	0	0	0	0	0	0	0	0	1.11	0	0	0	0	0	0	1	4.5	Firmicutes; Clostridia; Clostridiales;
Gemella haemolysans	AM157450	99	0	0	0	0	0	0	0	0	0	0	0	0	0	0	0	0	0	0	1.1	0	0	0	1	4.5	Firmicutes; Bacillales; Gemella.
Bacillus sp. L45	DQ248040	99	0	0	1.28	0	0	0	0	0	0	0	0	0	0	0	0	0	0	0	0	0	0	0	1	4.5	Firmicutes; Bacillales; Bacillaceae; Bacillus.
**Bacteroidetes**																											
Porphyromonas somerae	AY968205	89	0	0	0	0	0	0	0	0	0	0	0	0	0	0	0	47.78	0	0	0	0	0	0	43	4.5	Bacteroidetes; Bacteroidetes (class); Bacteroidales;
Flavobacterium sp. GPTSA100-9	AY904351	98	0	0	0	0	0	10.87	0	0	0	0	0	0	0	0	0	0	0	0	0	0	0	0	10	4.5	Bacteroidetes; Flavobacteria; Flavobacteriales;
Flavobacterium sp. MSY2	DQ196345	99	0	0	0	0	0	6.52	0	0	0	0	0	0	0	0	0	0	0	0	0	0	0	0	6	4.5	Bacteroidetes; Flavobacteria; Flavobacteriales;
Prevotella buccalis	L16476	98	0	0	0	0	0	0	0	0	0	0	0	0	0	3.49	3.37	0	0	0	0	0	0	0	6	9.1	Bacteroidetes; Bacteroidetes (class); Bacteroidales;
Prevotella sp. 4401737	DQ518919	99	0	0	0	0	0	0	0	0	0	0	0	0	0	0	0	3.33	0	0	0	0	0	0	3	4.5	Bacteroidetes; Bacteroidetes (class); Bacteroidales;
Cytophaga hutchinsonii	M58768	91	0	0	0	0	0	0	2.35	0	0	0	0	0	0	0	0	0	0	0	0	0	0	0	2	4.5	Bacteroidetes; Sphingobacteria; Sphingobacteriales;
Hymenobacter aerophilus	AJ276901	94	0	0	0	3.85	0	0	0	0	0	0	0	0	0	0	0	0	0	0	0	0	0	0	1	4.5	Bacteroidetes; Sphingobacteria; Sphingobacteriales;
Prevotella melaninogenica	AY323525	99	0	0	0	0	0	0	1.18	0	0	0	0	0	0	0	0	0	0	0	0	0	0	0	1	4.5	Bacteroidetes; Bacteroidetes (class); Bacteroidales;
Human oral bacterium C67	AF201974	99	0	0	0	0	0	0	1.18	0	0	0	0	0	0	0	0	0	0	0	0	0	0	0	1	4.5	Bacteroidetes; Bacteroidetes (class); Bacteroidales;
Porphyromonas endodontalis	AY253728	99	0	0	1.28	0	0	0	0	0	0	0	0	0	0	0	0	0	0	0	0	0	0	0	1	4.5	Bacteroidetes; Bacteroidetes (class); Bacteroidales;
Prevotellaceae bacterium P4P_62	AY207061	89	0	0	0	0	0	0	1.18	0	0	0	0	0	0	0	0	0	0	0	0	0	0	0	1	4.5	Bacteroidetes; Bacteroidetes (class); Bacteroidales;
Porphyromonas catoniae	X82823	94	0	0	0	0	0	0	1.18	0	0	0	0	0	0	0	0	0	0	0	0	0	0	0	1	4.5	Bacteroidetes; Bacteroidetes (class); Bacteroidales;
**Actinobacteria**																											
Mycobacterium intracellulare	DQ437715	99	0	0	17.95	0	0	0	0	0	0	0	0	0	0	0	0	0	0	0	0	0	0	0	14	4.5	Actinobacteria; Actinobacteridae; Actinomycetales;
Propionibacterium acnes	DQ672258	99	0	0	0	0	0	3.26	2.35	0	0	0	0	0	1.04	0	0	0	0	0	0	0	0	0	6	13.6	Actinobacteria; Actinobacteridae; Actinomycetales;
Mycobacterium coloregonium	AY624367	99	0	0	3.85	0	0	0	0	0	0	0	0	0	0	0	0	0	0	0	0	0	0	0	3	4.5	Actinobacteria; Actinobacteridae; Actinomycetales;
Microbacterium sp. RI48	DQ530139	98	0	0	1.28	0	0	0	0	0	0	0	0	0	0	0	0	0	0	0	0	0	0	0	1	4.5	Actinobacteria; Actinobacteridae; Actinomycetales;
Corynebacterium thomssenii	AF537607	100	0	0	0	0	1.69	0	0	0	0	0	0	0	0	0	0	0	0	0	0	0	0	0	1	4.5	Actinobacteria; Actinobacteridae; Actinomycetales;
Mycobacterium coloregonium	AY624367	99	0	0	1.28	0	0	0	0	0	0	0	0	0	0	0	0	0	0	0	0	0	0	0	1	4.5	Actinobacteria; Actinobacteridae; Actinomycetales;
Unknown Actinomycete (MC 9)	X68467	92	0	0	2.56	0	0	0	0	0	0	0	0	0	0	0	0	0	0	0	0	0	0	0	2	4.5	Actinobacteria; Actinobacteridae.
**Nitrospira**																											
Nitrospira marina	L35501	88	0	0	0	0	0	0	0	0	0	0	29.67	0	0	0	0	0	0	0	0	0	0	0	27	4.5	Nitrospirae; Nitrospirales; Nitrospiraceae; Nitrospira.
**Chloroplasts**																											
Zea mays chloroplast	NC_001666	99	0	0	0	3.85	0	0	0	0	0	0	0	0	0	0	0	0	0	0	0	0	0	0	1	4.5	Chloroplast
**Deinococci**																											
Deinococcus sp. 4B4	EU029131	98	0	0	1.28	0	0	0	0	0	0	0	0	0	0	0	0	0	0	0	0	0	0	0	1	4.5	Deinococcus-Thermus; Deinococci; Deinococcales;
**OP10**																											
Candidate division OP10 bacterium P488	AM749768	92	0	0	1.28	0	0	0	0	0	0	0	0	0	0	0	0	0	0	0	0	0	0	0	1	4.5	OP10.
**Planctomycetes**																											
Planctomycete str. 292	AJ231182	93	0	0	0	0	0	0	0	0	0	0	0	1.12	0	0	0	0	0	0	0	0	0	0	1	4.5	Planctomycetes; Planctomycetacia; Planctomycetales;
		Total Clones:	76	80	78	26	59	92	85	94	92	92	91	89	96	86	89	90	93	92	91	58	56	58	1763		
